# Probiotic *Lactiplantibacillus plantarum* Tana Isolated from an International Weightlifter Enhances Exercise Performance and Promotes Antifatigue Effects in Mice

**DOI:** 10.3390/nu14163308

**Published:** 2022-08-12

**Authors:** Mon-Chien Lee, Ming-Ju Chen, Hsiao-Wen Huang, Wei-Kai Wu, Yi-Wei Lee, Hsing-Chun Kuo, Chi-Chang Huang

**Affiliations:** 1 Graduate Institute of Sports Science, National Taiwan Sport University, Taoyuan City 33301, Taiwan; 2 Department of Animal Science and Technology, National Taiwan University, Taipei 106037, Taiwan; 3 Department of Medical Research, National Taiwan University Hospital, Taipei 106037, Taiwan; 4 Department of Internal Medicine, National Taiwan University Hospital, Taipei 106037, Taiwan; 5 Department of Physical Education, Fu-Jen Catholic University, New Taipei City 24205, Taiwan

**Keywords:** *Lactiplantibacillus plantarum*, probiotic, exercise, antifatigue

## Abstract

Exercise causes changes in the gut microbiota, and in turn, the composition of the gut microbiota affects exercise performance. In addition, the supplementation of probiotics is one of the most direct ways to change the gut microbiota. In recent years, the development and application of human-origin probiotics has gradually attracted attention. Therefore, we obtained intestinal *Lactiplantibacillus plantarum* “Tana” from a gold-medal-winning weightlifter, who has taken part in various international competitions such as the World Championships and the Olympic Games, to investigate the benefits of Tana supplementation for improving exercise performance and promoting antifatigue effects in mice. A total of 40 male Institute of Cancer Research (ICR) mice were divided into four groups (10 mice/group): (1) vehicle (0 CFU/mice/day), (2) Tana-1× (6.15 × 10^7^ CFU/mice/day), (3) Tana-2× (1.23 × 10^8^ CFU /mice/day), and (4) Tana-5× (3.09 × 10^8^ CFU/mice/day). After four weeks of Tana supplementation, we found that the grip strength, endurance exercise performance, and glycogen storage in the liver and muscle were significantly improved compared to those in the vehicle group (*p* < 0.05). In addition, supplementation with Tana had significant effects on fatigue-related biochemical markers; lactate, ammonia, and blood urea nitrogen (BUN) levels and creatine kinase (CK) activity were significantly lowered (*p* < 0.05). We also found that the improved exercise performance and antifatigue benefits were significantly dose-dependent on increasing doses of Tana supplementation (*p* < 0.05), which increased the abundance and ratio of beneficial bacteria in the gut. Taken together, Tana supplementation for four weeks was effective in improving the gut microbiota, thereby enhancing exercise performance, and had antifatigue effects. Furthermore, supplementation did not cause any physiological or histopathological damage.

## 1. Introduction

Human gut microbiota contains trillions of microbial cells from thousands of different species, as well as a variety of archaea, eukaryotic microorganisms, and viruses, with over three million genes and enormous metabolic capacity [1]. The normal human gut microbiota includes two major phyla, *Bacteroidetes* and *Firmicutes*, as well as other smaller phyla [2]. These microorganisms inhabiting the human gut are collectively known as the gut microbiota; they form a complex community, and their coexistence, synergy, and interaction with the host are considered critical to overall health and prevention of disease [3]. Many intrinsic and extrinsic environmental factors influence the composition of the gut microbiota, resulting in a highly dynamic and individualized complex gut ecosystem [4] that depends on important factors such as age, probiotic and antibiotic use, and diet [5]. In addition to this, exercise has also been recognized in recent years as one of the major behavioral factors that may affect the composition of the gut microbiota in animals and humans, which is dependent on the type, intensity, and duration of the exercise, as well as dietary habits and memory characteristics [6]. According to World Health Organization (WHO) guidelines, which recommend 150 min of moderate-intensity physical activity per week [7], the minimum level of physical activity appears to be sufficient to alter the composition of the gut microbiota [8]. In addition, research has shown that athletes have higher alpha diversity of gut bacteria than sedentary individuals [9]. This may be due to a higher protein intake by athletes, which increases the diversity of the gut microbiota, which in turn enhances tissue repair and utilization of dietary energy, while also increasing carbohydrate metabolism, cellular structure, and nucleotide biosynthesis ability [10]. Exercise causes changes in the gut microbiota, and conversely, the composition of the gut microbiota affects exercise performance. In the gut, microbiota composition and metabolic activity may contribute to fermentation and energy extraction from indigestible polysaccharides such as fiber and resistant starch, and conversion to short-chain fatty acids (SCFAs) such as acetate, propionate, and butyrate [11]. This has the effect of enhancing energy harvesting during exercise and provides metabolic benefits to athletes during high-intensity exercise and recovery [12]. Among the SCFAs, acetate and propionate are substrates for mitochondrial oxidation, lipid production, and gluconeogenesis, while propionate is a precursor for glucose synthesis in the liver [13]. In colonic epithelial cells, butyrate is mainly transported from cells to mitochondria as an energy source, and under aerobic conditions it is converted into acetyl-CoA through fatty acid oxidation (FAO), after which it enters the tricarboxylic acid cycle to generate NADH, and it then enters the electron transport chain to produce adenosine triphosphate (ATP) and CO_2_ [14]. Thus, SCFAs produced by the gut microbiota affect energy metabolism during exercise, thereby contributing to exercise-induced adaptation. SCFAs also act as an energy source for liver and muscle cells, improving endurance performance by keeping blood glucose constant [15].

Nutritional enhancers are commonly used to enhance athletic performance, enhance training adaptation, and improve exercise recovery [16]. In recent years, probiotics have been recognized as one of the most straightforward ways to beneficially alter and support the composition of the gut microbiota, and they are increasingly used by athletes as a sports nutrition supplement [17]. Probiotics are defined as live, non-pathogenic microorganisms that, when administered in sufficient amounts, confer a microbial balance and have a beneficial effect on the host [18]. Comprising many bacterial species, the most studied probiotics currently belong to the genera *Lactiplantibacillus* and *Bifidobacterium*. There is growing evidence that probiotic supplementation can improve exercise performance and fatigue recovery [19,20]. However, different strains may have different efficacies. In addition, the same bacteria from different sources may exhibit differences in intestinal colonization ability and even efficacy [21]. Common probiotics were previously derived primarily from fermented foods, such as yogurt, cheese, kimchi, miso, etc. [22]. In recent years, there has also been growing interest in the use of human probiotic strains, which are beneficial strains that live and thrive in the human digestive tract and contain no human by-products or ingredients [23]. In recent years, research has shown that human-origin probiotics have demonstrated superior results and performance in both in vitro and animal studies compared to plant- and dairy-derived probiotics [24]). Therefore, the development and application of human-origin probiotics have been receiving increasing attention [25].

Based on the above, we screened intestinal *Lactiplantibacillus* *plantarum* from Hsing-Chun Kuo, a gold-medal-winning weightlifter who has competed in various international competitions in recent years, such as the World Championships and the Olympic Games, and whose aboriginal name is Tana. We have reported in the past that *L. plantarum* derived from kimchi can effectively improve exercise performance, muscle mass, and fatigue resistance in animals and humans [20,26]. Therefore, in this study, we aimed to evaluate the benefits of humanized *L. plantarum* derived from the gut of Hsing-Chun Kuo (hereinafter called *L. plantarum* Tana) in improving exercise performance and exhibiting antifatigue effects in mice, and to further analyze changes in gut microbiota to explore related mechanisms.

## 2. Materials and Methods

### 2.1. Sample Preparation

*Lactiplantibacillus plantarum* subsp. *plantarum* was isolated from Hsing-Chun Kuo, gold medal winner of the 2020 Tokyo Olympics women’s 59 kg weightlifting competition. The fecal samples were aseptically taken in a sterile stool collection container and transported to the Department of Animal Science and Technology, National Taiwan University, Taipei, Taiwan, and immediately processed using the following procedure. Homogenized samples were used to perform serial 10-fold dilutions with saline and inoculated 0.1 mL aliquots onto MRS agar plates supplemented with 0.05% L-cysteine and 0.01% of both cycloheximide and sodium azide, and were then incubated anaerobically at 30 or 37 °C for 3 days. The colonies with distinct morphologies (e.g., in terms of size, color, and shape) were selected and purified by streaking at least three times on modified MRS agar plates. Genomic DNA of the LAB strains were extracted for identification purposes. The 16S rRNA gene was amplified with the primers 8F and 15R [27]. Phenylalanyl-tRNA synthase (pheS) and RNA polymerase A subunit (rpoA) genes were amplified with the primers pheS-21-F and pheS-23-R and rpoA-21-F and rpoA-23-R, respectively [28]. Full-length sequencing of the 16S rRNA gene was performed with the primers 350F, 520R, and 930F [29], and partial sequencing of the pheS and rpoA genes was performed with the previously described primers. All sequence analyses were carried out at Genomics BioSci & Tech Co., Ltd. (New Taipei, Taiwan). The sequences were assembled by using Chromas version 2.23 (Technelysium Pty. Ltd., South Brisbane, QLD, Australia), GENETYX version 5.1, and GENETYX ATSQ version 1.03 (Software Development Co., Tokyo, Japan). Phylogenetic trees were constructed by the neighbor-joining method [30] by using Clustal X software version 2.1 (Conway Institute, University College Dublin, Dublin, Ireland) [31]. The statistical reliability of the trees was evaluated by the bootstrap analysis of 1000 replicates [32] by using MEGA7 v7.0.14 software [33] according to Kimura’s two-parameter model as a substitution model [34]. *L. plantarum* is a Gram-positive lactic acid bacterium commonly found in fermented food and the gastrointestinal tract. It is also one of the widely used bacterial species in probiotic products. *L. plantarum* Tana were cultured in MRS broth at 37 °C for 24 h to reach the stationary phase and stored at −80 °C in the culture library at the Department of Animal Science and Technology, National Taiwan University, Taipei, Taiwan. Cells were centrifuged at 3300× *g* for 10 min and washed twice with saline. After the supernatant was discarded, cells were suspended in saline. The 1×, 2×, and 5× groups were orally gavaged with 200 µL of a cell suspension equal to 6.15 × 10^7^, 1.23 × 10^8^, and 3.09 × 10^8^ colony-forming units (CFUs)/mouse/day, respectively, for 4 weeks.

### 2.2. Experimental Design

**Figure 1:**
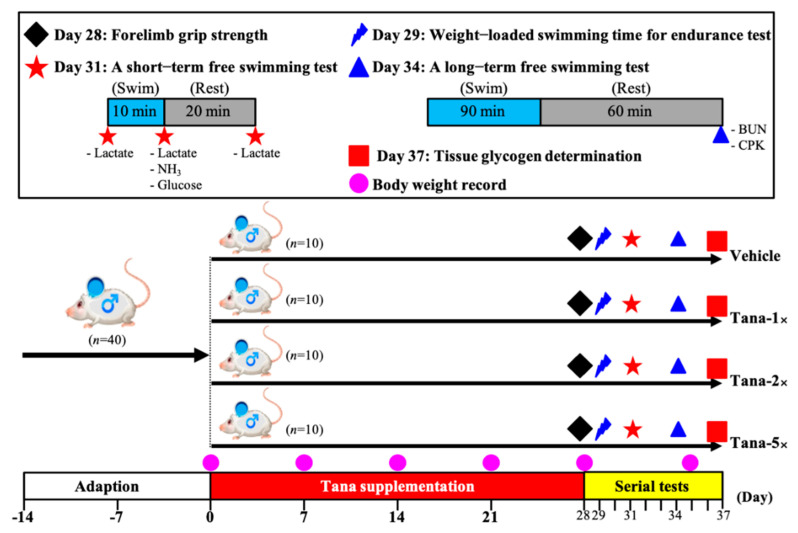
Experimental design.

Forty male Institute of Cancer Research (ICR) mice (6 weeks old) from BioLASCO Taiwan (Yi-Lan Breeding Center, Yi-Lan County, Taiwan) were used. This experiment was approved by the Institutional Animal Care and Use Committee (IACUC) of National Taiwan Sport University (IACUC-10914). All mice were given a standard laboratory diet (No. 5001; PMI Nutrition International, Brentwood, MO, USA) and distilled water ad libitum, and maintained in a 12-h light/12-h dark cycle, at room temperature (22 ± 2 °C) and 60–70% humidity. After being allowed food ad libitum for 2 weeks prior to experiments, the 40 mice were randomly assigned to four groups (10 mice/group) for oral gavage treatment for 4 weeks: (1) vehicle (0 CFUs/mouse/day), (2) Tana-1× (6.15 × 10^7^ CFUs/mouse/day), (3) Tana-2× (1.23 × 10^8^ CFUs/mouse/day), and (4) Tana-5× (3.09 × 10^8^ CFUs/mouse/day), and we recorded the body weight, water consumption, and food intake each week. The experimental flow chart is shown in Figure 1.

### 2.3. Forelimb Grip Strength

We used a low-force testing system (Model-RX-5, Aikoh Engineering, Nagoya, Japan) to measure the grip strength of mice undergoing vehicle or Tana supplementation. We gently held the mouse’s tail to allow it to swing naturally while the two front limbs of the mouse held a tension rod (diameter 2 mm, length 7.5 cm), then we pulled gently in the opposite direction, repeating 10 times. We recorded the maximum value through the force sensor [35].

### 2.4. Swimming Exercise Performance Test

As previously described [36], after 4 weeks of Tana intervention on day 29 for the swimming exhaustion test, weight loading 5% of each mouse’s body weight was attached to its tail, then mice were forced to swim in 27 ± 1 °C water until they lost coordinated movement or could not return to the surface within 7 s. We recorded the time from the beginning of the test until mouse exhaustion as the swimming endurance time.

### 2.5. Determination of Fatigue-Associated Serum Biomarkers

We evaluated the effect of Tana supplementation on biochemical markers and physiological states associated with post-exercise fatigue by swimming without weight loading; all mice were fasted for 12 h before each blood draw to reflect true physiological adaptation to exercise intervention. Day 31 after Tana intervention, serum lactate, ammonia (NH_3_), and glucose levels were ascertained before swimming and after 10 min of swimming and 20 min of rest by submandibular blood collection. On day 34 after Tana intervention, we also collected blood for analysis of blood urinary nitrogen (BUN) and creatine kinase (CK) after 90 min of swimming and 60 min of rest. Serum was collected from all blood samples by centrifugation at 1500× *g* for 15 min at 4 °C and was measured using an automatic analyzer (Hitachi, Tokyo, Japan, Hitachi 7060) [37].

### 2.6. Clinical Biochemical Profiles

On day 37 after 4 weeks of Tana intervention, thirty minutes after the last supplementation, all mice were euthanized by 95% CO_2_, and cardiac puncture blood collection was carried out immediately. After centrifugation to collect serum, the clinical biochemical variables, including aspartate aminotransferase (AST), alanine transaminase (ALT), albumin, triglycerides (TGs), blood urea nitrogen (BUN), creatinine, uric acid (UA), total protein (TP), CK, and glucose were measured using an autoanalyzer (model 717, Hitachi, Tokyo, Japan).

### 2.7. Visceral Tissue Weight, Histology Staining, and Glycogen Determination

After the mice were euthanized, we carefully removed, excised, and weighed the liver, kidneys, heart, lungs, muscles, epididymal fat pad (EFP), and brown adipose tissue (BAT). We stored parts of the muscle and liver tissues in liquid nitrogen for glycogen content analysis, as previously described [38].

### 2.8. Bacterial DNA Extraction and 16S rRNA Sequencing

After the mice were euthanized, the collected cecum content samples were immediately stored at −80 °C for DNA extraction. The sample extraction, preparation, and analysis proceeded according to the methods previously used in our laboratory [39].

### 2.9. Statistical Analysis

All data are expressed as mean ± SD for *n* = 10 mice per group, and the statistical analysis software used was SAS 9.0 (SAS Inst., Cary, NC, USA); we used one-way analysis of variance (ANOVA) to measure statistical differences among groups. The Cochran–Armitage test was used for the dose–effect trend analysis. *p* < 0.05 was considered statistically significant.

## 3. Results

### 3.1. Effect of Tana Supplementation on Grip Strength and Endurance Exercise Performance

**Figure 2:**
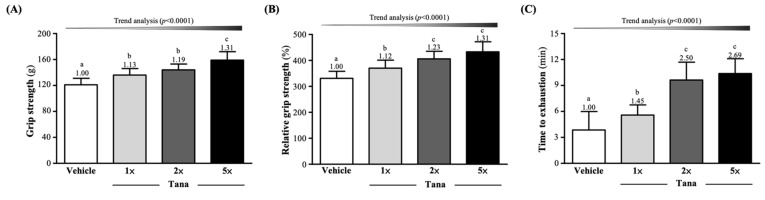
Effect of 4 weeks of Tana supplementation on (**A**) absolute forelimb grip strength, (**B**) relative forelimb grip strength, and (**C**) exhaustive swim time. Data are expressed as mean ± SD for *n* = 10 mice per group. Different superscript letters (a, b, c) indicate significant difference at *p* < 0.05.

As shown in Figure 2A, after four weeks of Tana supplementation, the absolute forelimb grip strengths in the vehicle, Tana-1×, Tana-2×, and Tana-5× groups were 121 ± 10, 136 ± 10, 144 ± 9, and 159 ± 13 (g), respectively. Compared with the vehicle group, the absolute grip strengths of the Tana-1×, Tana-2×, and Tana-5× groups increased significantly by 1.13-fold (*p* = 0.0025), 1.19-fold (*p* < 0.0001), and 1.31-fold (*p* < 0.0001), respectively. We normalized by mouse body weight to calculate relative grip strength (%) and found that the Tana supplementation group relative grip strengths were also significantly greater than the vehicle group (*p* < 0.05). The Tana supplementation had dose-dependent effects on absolute and relative grip strength (*p* < 0.0001) (Figure 2B).

The exhaustive swim times were tested and recorded after four weeks of Tana supplementation; the times for the vehicle, Tana-1×, Tana-2×, and Tana-5× groups were 3.86 ± 2.13, 5.58 ± 1.17, 9.63 ± 2.07, and 10.38 ± 1.71 min, respectively. The Tana-1×, Tana-2×, and Tana-5× group average exhaustive swim times were significantly increased compared to the vehicle group by 1.45-fold (*p* = 0.0404), 2.50-fold (*p* < 0.0001), and 2.69-fold (*p* < 0.0001), respectively. The effect of Tana supplementation on maximum swim time was dose-dependent (*p* < 0.0001) (Figure 2C).

### 3.2. Effect of Tana Supplementation on Serum Lactate Levels after the 10 min Swim Test

All the mice underwent a 10 min swimming and 20 min rest test to evaluate the levels of lactate after four weeks of supplementation with Tana (Table 1). There was no significant difference in lactate levels between groups before swimming. After 10 min of swimming, the serum lactate levels of mice in the vehicle, Tana-1×, Tana-2×, and Tana-5× groups were 7.45 ± 0.65, 6.79 ± 0.55, 6.41 ± 0.51, and 5.92 ± 0.59 mmol/L, respectively. The Tana-1×, Tana-2×, and Tana-5× group lactate levels were significantly decreased by 8.86% (*p* = 0.0152), 13.94% (*p* = 0.0003), and 20.56% (*p* < 0.0001), respectively. The lactate production rates were determined based on the serum lactate concentration before and after 10 min of swimming; in the vehicle, Tana-1×, Tana-2×, and Tana-5× groups the lactate production rates were 1.74 ± 0.04, 1.55 ± 0.07, 1.48 ± 0.16, and 1.35 ± 0.09, respectively. The Tana-2×, and Tana-5× group rates were significantly lower than those of the vehicle group by 14.97% (*p* = 0.0322) and 22.17% (*p* = 0.0019), respectively.

After 20 min rest following the swimming test, the vehicle, Tana-1×, Tana-2×, and Tana-5× group blood lactate levels were 6.20 ± 0.74, 5.38 ± 0.49, 5.12 ± 0.36, and 4.72 ± 0.24 mmol/L, respectively. Compared with the vehicle group, Tana-1×, Tana-2×, and Tana-5× group levels were significantly lower than those of the vehicle group by 13.19% (*p* = 0.0007), 17.34% (*p* < 0.0001), and 23.86% (*p* < 0.0001), respectively. However, there was no significant difference in the clearance rates between each group. The Tana supplementation had a dose-dependent effect on reduced serum lactate levels (*p* < 0.05).

### 3.3. Effect of Tana Supplementation on Fatigue-Related Indexes after the 10 min Swim test or a 90 min Swim Test and 60 min Rest

**Figure 3:**
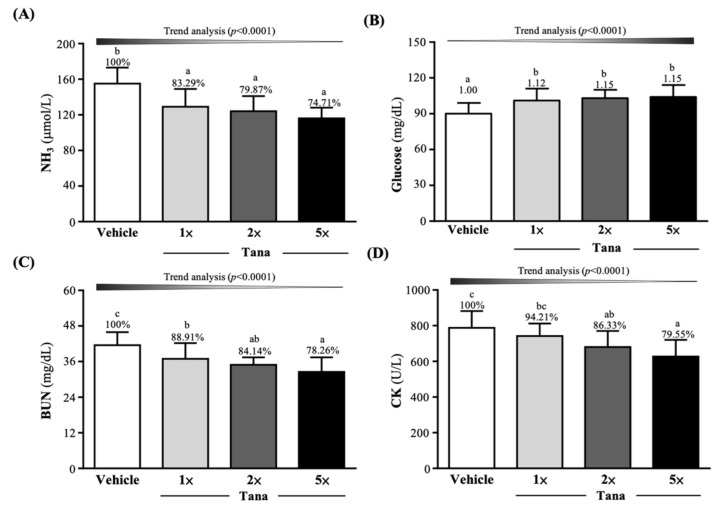
Effect of 4 weeks of Tana supplementation on (**A**) NH3, (**B**) glucose, (**C**) BUN, and (**D**) CK. Data are expressed as mean ± SD for *n* = 10 mice per group. Different superscript letters (a, b, c) indicate significant difference at *p* < 0.05.

After the 10 min swim test, the vehicle, Tana-1×, Tana-2×, and Tana-5× group serum ammonia levels were 155 ± 18, 129 ± 20, 124 ± 17, and 116 ± 12 µmol/L, respectively. The Tana-1×, Tana-2×, and Tana-5× group levels were significantly decreased by 16.71% (*p* = 0.0017), 20.13% (*p* = 0.0002), and 25.29% (*p* < 0.0001), respectively (Figure 3A). As shown in Figure 3B, the vehicle, Tana-1×, Tana-2×, and Tana-5× group glucose levels were 90 ± 9, 101 ± 10, 103 ± 7, and 104 ± 10 mg/dL, respectively. The Tana-1×, Tana-2×, and Tana-5× group levels were significantly higher than those of the vehicle group by 1.12-fold (*p* = 0.0120), 1.15-fold (*p* = 0.0018), and 1.15-fold (*p* = 0.0017). Supplementation with Tana decreased ammonia and improved glucose levels after exercise, and both had a dose-dependent effect (*p* < 0.0001).

As shown in Figure 3C, the serum BUN levels were measured 60 min after the 90 min swimming test; in the vehicle, Tana-1×, Tana-2×, and Tana-5× groups, the levels were 41.5 ± 4.4, 36.9 ± 5.3, 34.9 ± 2.5, and 32.5 ± 4.9 mg/dL, respectively. The Tana-1×, Tana-2×, and Tana-5× group levels significantly decreased compared to the vehicle group by 11.09% (*p* = 0.0262), 15.86% (*p* = 0.0021), and 21.74% (*p* < 0.0001), respectively. CK activity was used as an exercise injury index after the 90 min swim test and 60 min rest; in the vehicle, Tana-1×, Tana-2×, and Tana-5× groups, the CK activity indexes were 155 ± 18, 129 ± 20, 124 ± 17, and 116 ± 12 µmol/L, respectively. Compared with the vehicle group, the Tana-2×, and Tana-5× group indexes significantly decreased by 13.67% (*p* = 0.0092) and 20.45% (*p* = 0.0002), respectively (Figure 3D). The effect of Tana supplementation on serum BUN and CK levels was also dose-dependent (*p* < 0.0001).

### 3.4. Effect of Tana Supplementation on Liver and Muscle Glycogen

**Figure 4:**
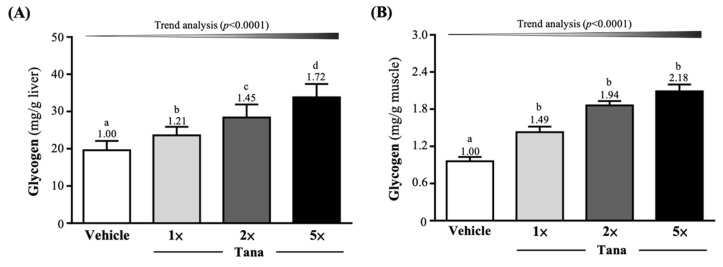
Effect of supplementation with Tana on (**A**) liver and (**B**) muscle. Data are expressed as mean ± SD for *n* = 10 mice per group. The different superscript letters (a, b, c, d) above each bar indicate a significant difference at *p* < 0.05.

The liver glycogen levels in the vehicle, Tana-1×, Tana-2×, and Tana-5× groups were 19.61 ± 2.49, 23.64 ± 2.26, 28.43 ± 3.47, and 33.81 ± 3.62 mg/g liver, respectively (Figure 4A). The Tana-1×, Tana-2×, and Tana-5× group levels were significantly improved by 1.21-fold (*p* = 0.0051), 1.45-fold (*p* < 0.0001), and 1.72-fold (*p* < 0.0001), respectively. As shown in Figure 4B, muscle glycogen levels in the vehicle, Tana-1×, Tana-2×, and Tana-5× groups were 0.96 ± 0.07, 1.43 ± 0.09, 1.86 ± 0.07, and 2.09 ± 0.11 mg/g muscle, respectively. Compared with the vehicle group, the Tana-1×, Tana-2×, and Tana-5× group levels were significantly increased by 1.49-fold (*p* < 0.0001), 1.94-fold (*p* < 0.0001), and 2.18-fold (*p* < 0.0001), respectively. Supplementation with Tana dose-dependently increased liver and muscle glycogen content (*p* < 0.0001).

### 3.5. General Characteristics of Mice with Tana Supplementation for Four Weeks

After four consecutive weeks of supplementation with Tana, for the weight, food intake, and water intake of each group, there was no significant difference between the groups, and the mice showed stable growth (Table 2). However, supplementation with Tana-5× significantly improved skeletal muscle mass and reduced EFP weight compared with the vehicle group by 1.10-fold (*p* = 0.0326) and 17.29% (*p* = 0.0477), respectively. Because tissue weight is affected by body weight differences, we divided the tissue weight by the relative percentage of body weight and found that, compared with the vehicle group, supplementation with Tana-5× significantly improved relative muscle mass by 1.10-fold (*p* = 0.0015) and reduced relative EFP weight by 17.74% (*p* = 0.0168). The effects of Tana supplementation on increased muscle weight and reduced EFP weight were dose-dependent (*p* < 0.05).

### 3.6. Effect of Tana Supplementation on Histopathology of Tissues and Biochemical Profiles at the End of the Study

**Figure 5:**
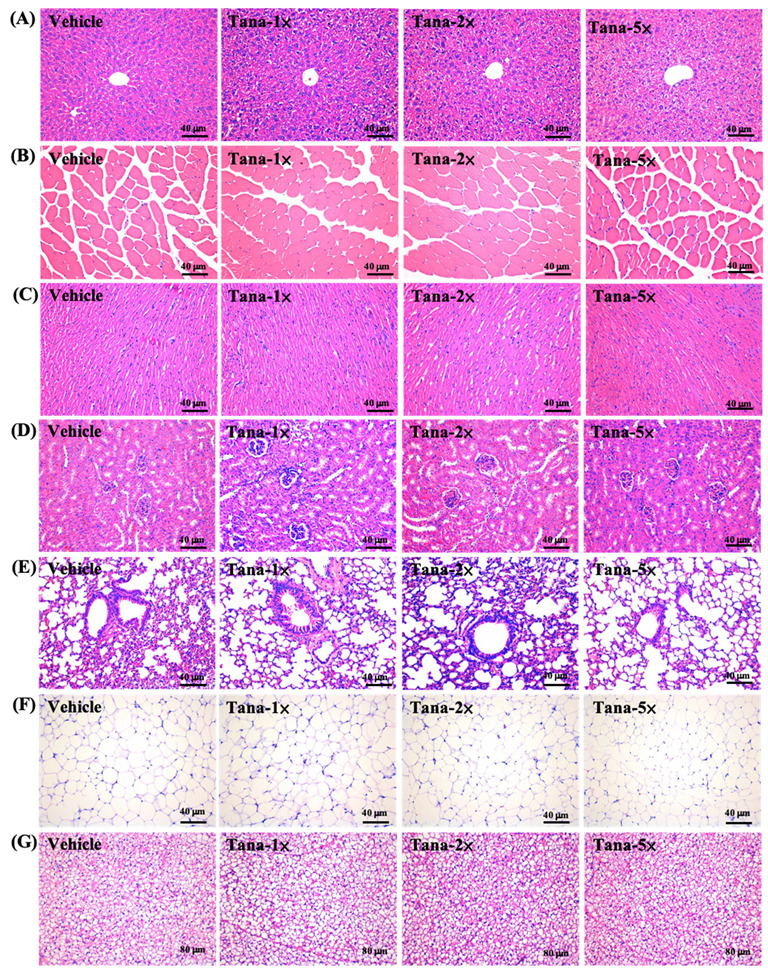
Effect of Tana supplementation on (**A**) liver, (**B**) kidney, (**C**) muscle, (**D**) heart, (**E**) lung, (**F**) adipocyte tissue, and (**G**) BAT tissue in mice. H&E stain, magnification: 200×; bar, 40 μm; BAT magnification: 100×; bar, 80 μm.

We assessed whether four weeks of Tana supplementation had effects on health and safety and tested the biochemical parameters (Table 3). We found that all biochemical parameters were within normal ranges, and there were no significant differences between each group. In addition, we performed histological evaluation of the liver, muscle, heart, kidney, lung, EFP, and BAT of mice, and no abnormalities or pathological changes were found in any group (Figure 5). Therefore, we believe that supplementation with Tana does not cause any harm.

### 3.7. Effect of Tana Supplementation on Gut Microbiota

**Figure 6:**
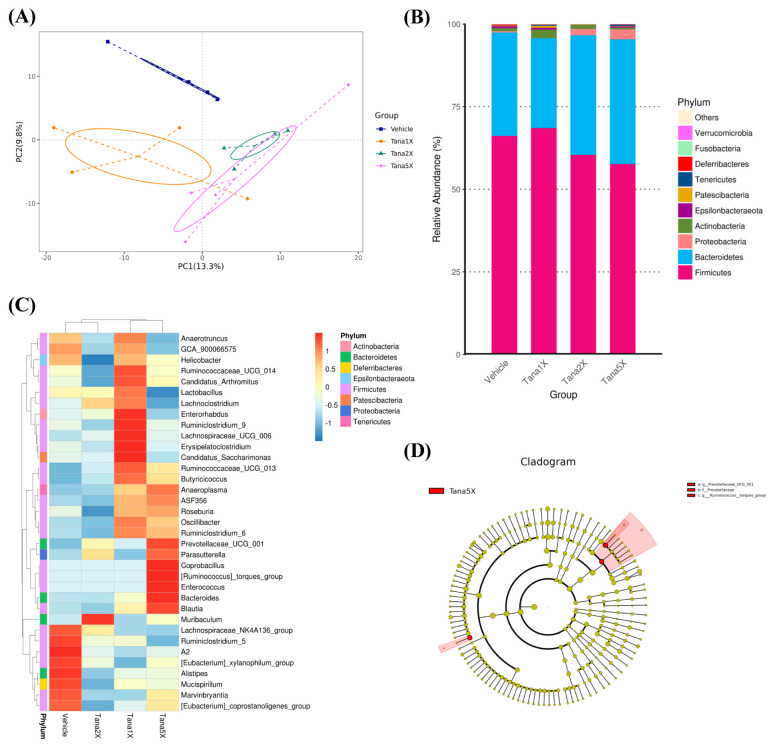
Effect of Tana supplementation: (**A**) effects of Tana supplementation on principal coordinate analysis (PCoA) of the gut microbiota composition in mice based on the Bray–Curtis distance measure of samples in the relative abundance profiles of an operational taxonomic unit; (**B**) effects of Tana supplementation on the phylum-level gut microbiota composition of mice. Only the top 10 phyla in average abundance are included; other phyla are classified as “Other”; (**C**) genus heatmap of gut microbiota; and (**D**) Tana-5× and vehicle group. Clade plots were generated from linear discriminant analysis effect size (LEfSe) analyses showing enrichment of the most differentially abundant taxa.

At the end of the experiment, we analyzed the gut microbiota composition of mice treated with vehicle, Tana-1×, Tana-2×, and Tana-5× through 16S rRNA and observed significant changes in microbial ecology after Tana treatment. As shown in Figure 6A, principal coordinate analysis of beta diversity using an unweighted UniFrac model demonstrated that mice clustered into relatively distinct groups according to different treatments, thus indicating that Tana significantly altered gut microbial populations. At the phylum level, *Firmicutes* accounted for the major proportion, with the vehicle, Tana-1×, Tana-2×, and Tana-5× groups accounting for 66%, 69%, 60%, and 58%, respectively, and *Bacteroidetes* accounting for 31%, 27%, 36%, and 38%, respectively. The *Firmicutes/Bacteroidetes* (F/B) ratios in the Tana-2× (1.78) and Tana-5× (1.82) groups, respectively, were lower than those in the vehicle (2.20) and Tana-1× (2.58) groups (Figure 6B). By observing the abundance of different bacterial genera between groups by heat map we found that, especially in the Tana-5× group, microorganisms beneficial to the gut microbiota such as *Prevotellaceae*, *Parasutterella*, *Ruminococcus*, and *Blautia* were more abundant (Figure 6C). We also confirmed by linear discriminant analysis effect size (LEfSe) that the number of *Prevotellaceae* in the Tana−5× group was higher than that in the vehicle group (Figure 6D).

## 4. Discussion

Exercise and training alter gut microbial abundance and expression. Supplementation with probiotics is one way to increase the number of good bacteria in the gut [1]. In recent years, athletes have increasingly chosen to use probiotics to regulate immunity, maintain healthy gastrointestinal function, and even increase energy utilization to improve exercise performance [40]. In the current study, we obtained *L*. *plantarum* Tana from one of the world’s top weightlifters and demonstrated in animal experiments that it can enhance exercise performance, improve body composition, and reduce exercise fatigue. We also observed changes in the composition of the gut microbiota. 

Probiotic supplements have been shown to alter the composition and metabolic activity of the gut microbiota, thereby promoting the growth of species that increase microbial diversity and health and assist in the conversion of indigestible carbohydrates into SCFAs [41]. Previous studies have indicated that probiotic supplementation with *Lactiplantibacillus* strains derived from the human gut could prevent diet-induced metabolic syndrome by modulating the gut microbiota and elevated acetate. This can improve energy harvesting during exercise, providing athletes with metabolic benefits during high-intensity exercise and recovery [42]. Butyrate is primarily used by epithelial cells in the colon as a source of energy, through conversion to acetyl-CoA, and it is used in the Krebs cycle to generate ATP [43]. In addition, butyrate can maintain blood glucose homeostasis and promote glycogen metabolism through the GPR43-AKT-GSK3 signaling pathway [44]. Propionate can be used as a precursor for glucose synthesis in the liver [45]. Previous studies indicated that administration of *Lactobacillus gasseri* SBT2055 to normal rats for four consecutive weeks reduced the area under the curve (AUC) for blood glucose concentration and increased the molar ratio of butyrate to total SCFA in the cecum [46]. In addition, *L. gasseri* SBT2055 was shown to ameliorate diabetes in rats by increasing glycogen storage in the liver and quadriceps and reducing serum-free fatty acid levels [47]. This appears to validate our results showing that 4 weeks of Tana supplementation significantly increased liver and muscle glycogen storage in mice (Figure 4A,B). Glycogen is mainly stored in the liver and muscles and is considered to be the main fuel source for long-term moderate-to-high-intensity endurance exercise [48]. When glycogen stores decrease to a certain level with increasing exercise intensity or duration, the exercise capacity of skeletal muscles is impaired, making it difficult to meet the energy demands of training and competition, resulting in increased fatigue, as well as decreased exercise capacity and endurance performance [49]. Furthermore, the modulation of SCFA production by the gut microbiota also directly affects energy metabolism during exercise and improves endurance performance through long-term maintenance of blood glucose [10]. Therefore, in this study, Tana supplementation had the effect of improving exercise endurance (Figure 2B). Similar to past studies, in a double-blind, crossover human trial, trained male runners supplemented with a multi-strain combination probiotic for 4 weeks experienced significantly longer fatigue time running in the heat compared to placebo; an increase of 16% [50]. In our past research, in addition to finding that supplementation with *L. plantarum* TWK10 can effectively improve the glycogen storage and endurance performance of mice, it had the effect of improving muscle strength and muscle mass. This may be related to the crosstalk pathway of the muscle–gut axis, which is affected by the composition and interaction of the microbiota and thus affects the mass, function, and energy metabolism of the muscle [51]. Muscle strength increases with muscle mass and is positively correlated [52]. Therefore, although Tana-5× supplementation alone was found to significantly improve muscle mass in this study (Table 2), 4 weeks of Tana supplementation without exercise training significantly improved grip performance (Figure 2A).

We also found that 4 weeks of Tana supplementation altered the gut microbiome, increasing the abundance and proportion of *Prevotellaceae* in the mouse gut, especially in the Tana-5× group (Figure 6A). A past study investigating the guts of long-term exercise cyclists found not only an increased abundance of *Akkermansia*, a bacterium commonly found in the guts of athletes, but also observed that the abundance of *Prevotella* was associated with exercise training [53]. *Prevotella* is thought to promote better glucose tolerance and glycogen storage in mice [54] and might be the key flora for improving glycogen storage and endurance performance in this study. In addition, the increased abundance of *Prevotella* was positively associated with many amino acid and carbohydrate metabolic pathways, including the metabolic branched-chain amino acids (BCAAs) leucine, isoleucine, and valine [53]. High levels of BCAAs can reduce exercise-induced central fatigue and muscle fatigue, and promote muscle protein synthesis [55], thereby reducing muscle damage during prolonged exercise [35]. This appears to explain the benefit of reduced post-exercise CK activity in the Tana group following exercise (Figure 3D). Additionally, lactate, NH_3_, and BUN are thought to reduce exercise performance and contribute to fatigue byproducts as exercise duration or intensity increases [56]. Among them, lactate is the product of carbohydrate glycolysis under anaerobic conditions, and during vigorous exercise, glucose is broken down into pyruvate. Under anaerobic conditions, hydrogen ions are reduced and released, resulting in lower blood and muscle tissue pH and inhibition of glycolysis, which interferes with normal cellular function [57]. In addition, when amino acid metabolism produces ATP for energy, ammonia is produced and accumulated in skeletal muscle, which may affect central fatigue [58]. At this point, ammonia is converted to BUN by the urea cycle, which is either increased in the blood or excreted in the urine [59]. However, a past study that obtained *Veillonella* from the guts of marathon runners and administered it to mice found that when systemic lactate produced during exercise entered the gut lumen, *Veillonella*, via the methylmalonyl-CoA pathway, converts the lactate to SCFAs, especially propionate, to improve exercise performance [60]. Additionally, past research has shown that probiotics can increase the hepatic clearance of ammonia and other toxins by reducing inflammation and oxidative stress in liver cells [61]. These seem to be plausible explanations, and in this study, Tana supplementation was effective in reducing post-exercise increases in blood lactate, NH_3_, and BUN (Figure 3A–C).

In the current study, in addition to exploring the benefits of Tana supplementation on exercise performance and antifatigue effects, we further confirmed through histopathological interpretation (Figure 5) and blood analysis (Table 3) that Tana supplementation did not cause any adverse effects. In addition to increasing muscle mass in terms of tissue weight, Tana supplementation also exhibited a body-fat reduction benefit, especially in the Tana-5× group (Table 2). This may be related to the F/B ratio [62]. Past research has shown that exercise training in mice increases *Bacteroidetes* while reducing *Firmicutes*, suggesting that exercise plays an important role in preventing diet-induced obesity, similar to the microbiome of lean mice [63]. This is similar to the results of this study, where higher doses of Tana supplementation alone appeared to reduce the F/B ratio in the absence of exercise training (Figure 6B). However, there is disagreement about whether the F/B ratio should be high or low, although the balance should be optimal. Previous studies have suggested that the F/B ratio is related to exercise performance, but the supplemented *L. plantarum* in our study resulted in higher *Prevotella* levels in the gut, which requires further study to explore the possible mechanism.

In sum, in this study, we have shown that four consecutive weeks of *L. plantarum* Tana supplementation can effectively improve exercise performance, glycogen storage, and muscle mass in mice, as well as reducing the production of post-exercise fatigue products. In addition, it did not cause any damage to the health of the mice. However, there are still relatively few studies using human probiotics for athletic performance, even from the guts of top athletes. In this study, in addition to the efficacy evaluation, it was also necessary to explore whether supplementation caused adverse effects, so animal experiments were first conducted. In the future, it will be necessary to further explore the mechanism of action to improve exercise performance, and to verify the evaluation of human intestinal colonization ability and benefit through human trials. It will also be necessary to explore whether supplementation has an additive benefit with exercise training, and the possibility of Tana being used as a sports nutrition supplement.

## 5. Conclusions

In the present study, we found that 4 weeks of Tana supplementation significantly increased glycogen storage, forelimb grip strength, and endurance performance in mice and significantly decreased levels of fatigue markers such as lactate, BUN, ammonia, and CK. This may be related to Tana altering the gut microbiota, thereby promoting host metabolic phenotypes. Therefore, Tana has proven useful as a supplement to improve exercise performance and reduce fatigue. Further research should be conducted in the future to determine the molecular mechanism of Tana involvement in antifatigue and to conduct human clinical trials.

## Figures and Tables

**Table 1:** Effect of Tana on lactate levels.

	Groups	Vehicle	Tana-1×	Tana-2×	Tana-5×	Trend Analysis
Time Point		Lactate (mmol/L)				
Before swimming (A)		4.33 ± 0.66 ^a^	4.38 ± 0.36 ^a^	4.39 ± 0.67 ^a^	4.40 ± 0.62 ^a^	0.9567
After swimming (B)		7.45 ± 0.65 ^c^	6.79 ± 0.55 ^b^	6.41 ± 0.51 ^ab^	5.92 ± 0.59 ^a^	<0.0001 *
After a 20 min resting (C)		6.20 ± 0.74 ^c^	5.38 ± 0.49 ^b^	5.12 ± 0.36 ^ab^	4.72 ± 0.24 ^a^	<0.0001 *
		Rates of lactate production and clearance				
Production rate = B/A		1.74 ± 0.13 ^b^	1.55 ± 0.07 ^ab^	1.48 ± 0.16 ^a^	1.35 ± 0.08 ^a^	0.0003 *
Clearance rate = (B − C)/B		0.17 ± 0.04 ^a^	0.21 ± 0.03 ^a^	0.20 ± 0.02 ^a^	0.20 ± 0.04 ^a^	0.5448

Lactate production rate (B/A) was the value of the lactate level after exercise (B) divided by that before exercise (A). Clearance rate (B − C)/B was defined as lactate level after swimming (B) minus that after 20 min rest (C) divided by that after swimming (B). Data are expressed as mean ± SD (*n* = 10 mice per group). Values in the same row with different superscript letters (a, b, c) differ significantly, *p* < 0.05. * Indicated significant dose dependence.

**Table 2:** Effect of Tana supplementation on various parameters.

Characteristics	Vehicle	Tana-1×	Tana-2×	Tana-5×	Trend Analysis
Initial BW (g)	34.7 ± 1.1 ^a^	34.8 ± 1.3 ^a^	34.7 ± 1.5 ^a^	34.7 ± 1.1 ^a^	0.9693
Final BW (g)	38.3 ± 1.3	38.5 ± 2.2 ^a^	38.2 ± 0.8 ^a^	38.4 ± 1.7 ^a^	0.8048
Water intake (mL/mouse/day)	9.3 ± 1.8 ^a^	9.4 ± 2.1 ^a^	9.4 ± 1.3 ^a^	9.3 ± 1.6 ^a^	0.4960
Diet intake (g/mouse/day)	7.4 ± 1.4 ^a^	7.3 ± 1.1 ^a^	7.2 ± 1.6 ^a^	7.2 ± 1.1 ^a^	0.2722
Liver (g)	2.22 ± 0.09 ^a^	2.24 ± 0.07 ^a^	2.21 ± 0.06 ^a^	2.23 ± 0.10 ^a^	0.7482
Muscle (g)	0.36 ± 0.01 ^a^	0.38 ± 0.06 ^ab^	0.38 ± 0.02 ^ab^	0.40 ± 0.03 ^b^	0.0048 *
Kidney (g)	0.63 ± 0.06 ^a^	0.64 ± 0.05 ^a^	0.63 ± 0.04 ^a^	0.63 ± 0.04 ^a^	0.8507
Heart (g)	0.20 ± 0.02 ^a^	0.20 ± 0.02 ^a^	0.20 ± 0.02 ^a^	0.20 ± 0.02 ^a^	0.4716
Lung (g)	0.23 ± 0.01 ^a^	0.23 ± 0.02 ^a^	0.23 ± 0.02 ^a^	0.23 ± 0.01 ^a^	0.9368
EFP (g)	0.35 ± 0.08 ^b^	0.34 ± 0.07 ^ab^	0.34 ± 0.03 ^ab^	0.29 ± 0.06 ^a^	0.0593
BAT (g)	0.11 ± 0.03 ^a^	0.10 ± 0.02 ^a^	0.11 ± 0.02 ^a^	0.11 ± 0.03 ^a^	0.8057
Cecum (g)	1.02 ± 0.11 ^a^	1.03 ± 0.19	1.04 ± 0.11 ^a^	1.13 ± 0.13 ^a^	0.0542
Relative liver weight (%)	5.80 ± 0.07 ^a^	5.82 ± 0.18 ^a^	5.79 ± 0.05 ^a^	5.81 ± 0.11 ^a^	0.9332
Relative muscle weight (%)	0.95 ± 0.01 ^a^	0.99 ± 0.09 ^ab^	0.99 ± 0.03 ^ab^	1.04 ± 0.05 ^b^	<0.0001 *
Relative kidney weight (%)	1.64 ± 0.10 ^a^	1.65 ± 0.06 ^a^	1.65 ± 0.08 ^a^	1.65 ± 0.03 ^a^	0.8840
Relative heart weight (%)	0.53 ± 0.05 ^a^	0.52 ± 0.05 ^a^	0.52 ± 0.04 ^a^	0.52 ± 0.04 ^a^	0.6946
Relative lung weight (%)	0.59 ± 0.03 ^a^	0.59 ± 0.03 ^a^	0.59 ± 0.03 ^a^	0.59 ± 0.04 ^a^	0.6745
Relative EFP weight (%)	0.90 ± 0.19 ^b^	0.88 ± 0.15 ^b^	0.88 ± 0.07 ^b^	0.74 ± 0.13 ^a^	0.0182 *
Relative BAT weight (%)	0.28 ± 0.06 ^a^	0.26 ± 0.03 ^a^	0.27 ± 0.05 ^a^	0.28 ± 0.06 ^a^	0.7992
Relative cecum weight (%)	2.66 ± 0.21 ^a^	2.65 ± 0.35 ^a^	2.71 ± 0.23 ^a^	2.95 ± 0.21 ^b^	0.0054 *

Data are expressed as mean ± SD (*n* = 10 mice per group). Values in the same row with different superscript letters (a, b) differ significantly, *p* < 0.05. EFP, epididymal fat paid; BAT, brown adipose tissue. * Indicated significant dose dependence.

**Table 3:** Effect of Tana supplementation on biochemical parameters.

Parameter	Vehicle	Tana-1×	Tana-2×	Tana-5×	Trend Analysis
AST (U/L)	93 ± 9 ^a^	92 ± 8 ^a^	93 ± 10 ^a^	93 ± 10 ^a^	0.6056
ALT (U/L)	55 ± 10 ^a^	52 ± 10 ^a^	52 ± 8 ^a^	52 ± 11 ^a^	0.6537
CK (U/L)	250 ± 45 ^a^	230 ± 43 ^a^	229 ± 33 ^a^	224 ± 33 ^a^	0.3291
GLU (mg/dL)	182 ± 20 ^a^	181 ± 24 ^a^	181 ± 29 ^a^	181 ± 33 ^a^	0.7611
CREA (mg/dL)	0.41 ± 0.03 ^a^	0.41 ± 0.02 ^a^	0.40 ± 0.05 ^a^	0.40 ± 0.03 ^a^	0.4376
BUN (mg/dL)	25.6 ± 2.4 ^a^	25.8 ± 1.4 ^a^	25.7 ± 1.4 ^a^	25.5 ± 1.8 ^a^	0.7100
UA (mg/dL)	2.4 ± 0.6 ^a^	2.6 ± 0.6 ^a^	2.6 ± 0.5 ^a^	2.5 ± 0.6 ^a^	0.8459
TC (mg/dL)	143 ± 14 ^a^	144 ± 17 ^a^	147 ± 11 ^a^	144 ± 11 ^a^	0.5140
TG (mg/dL)	166 ± 24 ^a^	164 ± 26 ^a^	167 ± 16 ^a^	162 ± 18 ^a^	0.5624
ALB (g/dL)	3.5 ± 0.1 ^a^	3.5 ± 0.1 ^a^	3.5 ± 0.1 ^a^	3.5 ± 0.2 ^a^	0.5982
TP (g/dL)	6.0 ± 0.2 ^a^	6.1 ± 0.1 ^a^	6.0 ± 0.2 ^a^	6.1 ± 0.3 ^a^	0.1561

Data are expressed as mean ± SD (*n* = 10 mice per group). AST, aspartate aminotransferase; ALT, alanine transaminase; ALB, albumin; BUN, blood urea nitrogen; CREA, creatinine; UA, uric acid; TP, total protein; TG, triacylglycerol; CK, creatine kinase. The same superscript letters (a) above each bar indicate with no difference at *p* > 0.05.

## Data Availability

The data presented in this study are available within the article.

## References

[1] Mohr A.E., Jäger R., Carpenter K.C., Kerksick C.M., Purpura M., Townsend J.R., West N.P., Black K., Gleeson M., Pyne D.B. (2020). The athletic gut microbiota. J. Int. Soc. Sports Nutr..

[2] Jandhyala S.M., Talukdar R., Subramanyam C., Vuyyuru H., Sasikala M., Nageshwar Reddy D. (2015). Role of the normal gut microbiota. World J. Gastroenterol..

[3] Milani C., Duranti S., Bottacini F., Casey E., Turroni F., Mahony J., Belzer C., Delgado Palacio S., Arboleya Montes S., Mancabelli L. (2017). The First Microbial Colonizers of the Human Gut: Composition, Activities, and Health Implications of the Infant Gut Microbiota. Microbiol. Mol. Biol. Rev..

[4] Duffy L.C., Raiten D.J., Hubbard V.S., Starke-Reed P. (2015). Progress and challenges in developing metabolic footprints from diet in human gut microbial cometabolism. J. Nutr..

[5] Sakkas H., Bozidis P., Touzios C., Kolios D., Athanasiou G., Athanasopoulou E., Gerou I., Gartzonika C. (2020). Nutritional Status and the Influence of the Vegan Diet on the Gut Microbiota and Human Health. Medicina.

[6] Allen J.M., Mailing L.J., Niemiro G.M., Moore R., Cook M.D., White B.A., Holscher H.D., Woods J.A. (2018). Exercise Alters Gut Microbiota Composition and Function in Lean and Obese Humans. Med. Sci. Sports Exerc..

[7] World Health Organization (2010). Global Recommendations on Physical Activity for Health.

[8] Bressa C., Bailén-Andrino M., Pérez-Santiago J., González-Soltero R., Pérez M., Montalvo-Lominchar M.G., Maté-Muñoz J.L., Domínguez R., Moreno D., Larrosa M. (2017). Differences in gut microbiota profile between women with active lifestyle and sedentary women. PLoS ONE.

[9] Clarke S.F., Murphy E.F., O’Sullivan O., Lucey A.J., Humphreys M., Hogan A., Hayes P., O’Reilly M., Jeffery I.B., Wood-Martin R. (2014). Exercise and associated dietary extremes impact on gut microbial diversity. Gut.

[10] Barton W., Penney N.C., Cronin O., Garcia-Perez I., Molloy M.G., Holmes E., Shanahan F., Cotter P.D., O’Sullivan O. (2018). The microbiome of professional athletes differs from that of more sedentary subjects in composition and particularly at the functional metabolic level. Gut.

[11] Pascale A., Marchesi N., Marelli C., Coppola A., Luzi L., Govoni S., Giustina A., Gazzaruso C. (2018). Microbiota and metabolic diseases. Endocrine.

[12] Okamoto T., Morino K., Ugi S., Nakagawa F., Lemecha M., Ida S., Ohashi N., Sato D., Fujita Y., Maegawa H. (2019). Microbiome potentiates endurance exercise through intestinal acetate production. Am. J. Physiol. Endocrinol. Metab..

[13] Nicholson J.K., Holmes E., Kinross J., Burcelin R., Gibson G., Jia W., Pettersson S. (2012). Host-gut microbiota metabolic interactions. Science.

[14] Donohoe D.R., Garge N., Zhang X., Sun W., O’Connell T.M., Bunger M.K., Bultman S.J. (2011). The microbiome and butyrate regulate energy metabolism and autophagy in the mammalian colon. Cell Metab..

[15] Tung Y.T., Chen Y.J., Chuang H.L., Huang W.C., Lo C.T., Liao C.C., Huang C.C. (2017). Characterization of the serum and liver proteomes in gut-microbiota-lacking mice. Int. J. Med. Sci..

[16] Kerksick C.M., Wilborn C.D., Roberts M.D., Smith-Ryan A., Kleiner S.M., Jäger R., Collins R., Cooke M., Davis J.N., Galvan E. (2018). ISSN exercise & sports nutrition review update: Research & recommendations. J. Int. Soc. Sports Nutr..

[17] Lamprecht M., Bogner S., Schippinger G., Steinbauer K., Fankhauser F., Hallstroem S., Schuetz B., Greilberger J.F. (2012). Probiotic supplementation affects markers of intestinal barrier, oxidation, and inflammation in trained men; a randomized, double-blinded, placebo-controlled trial. J. Int. Soc. Sports Nutr..

[18] Williams N.T. (2010). Probiotics. Am. J. Health Syst. Pharm..

[19] Lee M.C., Hsu Y.J., Chuang H.L., Hsieh P.S., Ho H.H., Chen W.L., Chiu Y.S., Huang C.C. (2019). In Vivo Ergogenic Properties of the Bifidobacterium longum OLP-01 Isolated from a Weightlifting Gold Medalist. Nutrients.

[20] Huang W.C., Lee M.C., Lee C.C., Ng K.S., Hsu Y.J., Tsai T.Y., Young S.L., Lin J.S., Huang C.C. (2019). Effect of Lactobacillus plantarum TWK10 on Exercise Physiological Adaptation, Performance, and Body Composition in Healthy Humans. Nutrients.

[21] Pyne D.B., West N.P., Cox A.J., Cripps A.W. (2015). Probiotics supplementation for athletes—Clinical and physiological effects. Eur. J. Sport Sci..

[22] George Kerry R., Patra J.K., Gouda S., Park Y., Shin H.S., Das G. (2018). Benefaction of probiotics for human health: A review. J. Food Drug Anal..

[23] Versalovic J. (2013). The human microbiome and probiotics: Implications for pediatrics. Ann. Nutr. Metab..

[24] Vemuri R., Shinde T., Shastri M.D., Perera A.P., Tristram S., Martoni C.J., Gundamaraju R., Ahuja K.D.K., Ball M., Eri R. (2018). A human origin strain Lactobacillus acidophilus DDS-1 exhibits superior in vitro probiotic efficacy in comparison to plant or dairy origin probiotics. Int. J. Med. Sci..

[25] Nagpal R., Wang S., Ahmadi S., Hayes J., Gagliano J., Subashchandrabose S., Kitzman D.W., Becton T., Read R., Yadav H. (2018). Human-origin probiotic cocktail increases short-chain fatty acid production via modulation of mice and human gut microbiome. Sci. Rep..

[26] Chen Y.M., Wei L., Chiu Y.S., Hsu Y.J., Tsai T.Y., Wang M.F., Huang C.C. (2016). Lactobacillus plantarum TWK10 Supplementation Improves Exercise Performance and Increases Muscle Mass in Mice. Nutrients.

[27] Watanabe K., Fujimoto J., Sasamoto M., Dugersuren J., Tumursuh T., Demberel S. (2008). Diversity of lactic acid bacteria and yeasts in Airag and Tarag, traditional fermented milk products of Mongolia. World J. Microbio. Biotechnol..

[28] Naser S.M., Thompson F.L., Hoste B., Gevers D., Dawyndt P., Vancanneyt M., Swings J. (2005). Application of multilocus sequence analysis (MLSA) for rapid identification of Enterococcus species based on rpoA and pheS genes. Microbiology.

[29] Miyake T., Watanabe K., Watanabe T., Oyaizu H. (1998). Phylogenetic analysis of the genus Bifidobacterium and related genera based on 16S rDNA sequences. Microbiol. Immunol..

[30] Saitou N., Nei M. (1987). The neighbor-joining method: A new method for reconstructing phylogenetic trees. Mol. Biol. Evol..

[31] Thompson J.D., Gibson T.J., Plewniak F., Jeanmougin F., Higgins D.G. (1997). The CLUSTAL_X windows interface: Flexible strategies for multiple sequence alignment aided by quality analysis tools. Nucleic Acids Res..

[32] Felsenstein J. (1985). Confidence limits on phylogenies: An approach using the bootstrap. Evolution.

[33] Kumar S., Stecher G., Tamura K. (2016). MEGA7: Molecular evolutionary genetics analysis version 7.0 for bigger datasets. Mol. Biol. Evol..

[34] Kimura M. (1980). A simple method for estimating evolutionary rates of base substitutions through comparative studies of nucleotide sequences. J. Mol. Evol..

[35] Lee M.C., Hsu Y.J., Lin Y.Q., Chen L.N., Chen M.T., Huang C.C. (2022). Effects of Perch Essence Supplementation on Improving Exercise Performance and Anti-Fatigue in Mice. Int. J. Environ. Res. Public Health.

[36] Ho C.S., Tung Y.T., Kung W.M., Huang W.C., Leung W.K., Huang C.C., Wu J.H. (2017). Effect of Coriolus versicolor Mycelia Extract on Exercise Performance and Physical Fatigue in Mice. Int. J. Med. Sci..

[37] Huang S.W., Hsu Y.J., Lee M.C., Li H.S., Yeo P.C.W., Lim A.L., Huang C.C. (2018). In Vitro and In Vivo Functional Characterization of Essence of Chicken as An Ergogenic Aid. Nutrients.

[38] Yeh W.L., Hsu Y.J., Ho C.S., Ho H.H., Kuo Y.W., Tsai S.Y., Huang C.C., Lee M.C. (2022). Lactobacillus plantarum PL-02 Supplementation Combined With Resistance Training Improved Muscle Mass, Force, and Exercise Performance in Mice. Front. Nutr..

[39] Hsu Y.J., Huang W.C., Lin J.S., Chen Y.M., Ho S.T., Huang C.C., Tung Y.T. (2018). Kefir Supplementation Modifies Gut Microbiota Composition, Reduces Physical Fatigue, and Improves Exercise Performance in Mice. Nutrients.

[40] Jäger R., Mohr A.E., Carpenter K.C., Kerksick C.M., Purpura M., Moussa A., Townsend J.R., Lamprecht M., West N.P., Black K. (2019). International Society of Sports Nutrition Position Stand: Probiotics. J. Int. Soc. Sports Nutr..

[41] Sánchez B., Delgado S., Blanco-Míguez A., Lourenço A., Gueimonde M., Margolles A. (2017). Probiotics, gut microbiota, and their influence on host health and disease. Mol. Nutr Food Res..

[42] Marttinen M., Ala-Jaakkola R., Laitila A., Lehtinen M.J. (2020). Gut Microbiota, Probiotics and Physical Performance in Athletes and Physically Active Individuals. Nutrients.

[43] LeBlanc J.G., Chain F., Martín R., Bermúdez-Humarán L.G., Courau S., Langella P. (2017). Beneficial effects on host energy metabolism of short-chain fatty acids and vitamins produced by commensal and probiotic bacteria. Microb. Cell Fact..

[44] Zhang W.Q., Zhao T.T., Gui D.K., Gao C.L., Gu J.L., Gan W.J., Huang W., Xu Y., Zhou H., Chen W.N. (2019). Sodium Butyrate Improves Liver Glycogen Metabolism in Type 2 Diabetes Mellitus. J. Agric. Food Chem..

[45] Samuel B.S., Shaito A., Motoike T., Rey F.E., Backhed F., Manchester J.K., Hammer R.E., Williams S.C., Crowley J., Yanagisawa M. (2008). Effects of the gut microbiota on host adiposity are modulated by the short-chain fatty-acid binding G protein-coupled receptor, Gpr41. Proc. Natl. Acad. Sci. USA.

[46] Shirouchi B., Nagao K., Umegatani M., Shiraishi A., Morita Y., Kai S., Yanagita T., Ogawa A., Kadooka Y., Sato M. (2016). Probiotic Lactobacillus gasseri SBT2055 improves glucose tolerance and reduces body weight gain in rats by stimulating energy expenditure. Br. J. Nutr..

[47] Niibo M., Shirouchi B., Umegatani M., Morita Y., Ogawa A., Sakai F., Kadooka Y., Sato M. (2019). Probiotic Lactobacillus gasseri SBT2055 improves insulin secretion in a diabetic rat model. J. Dairy Sci..

[48] Tsintzas K., Williams C. (1998). Human muscle glycogen metabolism during exercise. Effect of carbohydrate supplementation. Sports Med..

[49] Bergström J., Hultman E. (1967). A study of the glycogen metabolism during exercise in man. Scand. J. Clin. Lab. Investig..

[50] Shing C.M., Peake J.M., Lim C.L., Briskey D., Walsh N.P., Fortes M.B., Ahuja K.D., Vitetta L. (2014). Effects of probiotics supplementation on gastrointestinal permeability, inflammation and exercise performance in the heat. Eur. J. Appl. Physiol..

[51] Grosicki G.J., Fielding R.A., Lustgarten M.S. (2018). Gut Microbiota Contribute to Age-Related Changes in Skeletal Muscle Size, Composition, and Function: Biological Basis for a Gut-Muscle Axis. Calcif. Tissue Int..

[52] Chen L., Nelson D.R., Zhao Y., Cui Z., Johnston J.A. (2013). Relationship between muscle mass and muscle strength, and the impact of comorbidities: A population-based, cross-sectional study of older adults in the United States. BMC Geriatr..

[53] Petersen L.M., Bautista E.J., Nguyen H., Hanson B.M., Chen L., Lek S.H., Sodergren E., Weinstock G.M. (2017). Community characteristics of the gut microbiomes of competitive cyclists. Microbiome.

[54] Kovatcheva-Datchary P., Nilsson A., Akrami R., Lee Y.S., De Vadder F., Arora T., Hallen A., Martens E., Björck I., Bäckhed F. (2015). Dietary Fiber-Induced Improvement in Glucose Metabolism Is Associated with Increased Abundance of Prevotella. Cell Metab..

[55] Negro M., Giardina S., Marzani B., Marzatico F. (2008). Branched-chain amino acid supplementation does not enhance athletic performance but affects muscle recovery and the immune system. J. Sports Med. Phys. Fitness..

[56] Halson S.L., Bridge M.W., Meeusen R., Busschaert B., Gleeson M., Jones D.A., Jeukendrup A.E. (2002). Time course of performance changes and fatigue markers during intensified training in trained cyclists. J. Appl. Physiol..

[57] Proia P., Di Liegro C.M., Schiera G., Fricano A., Di Liegro I. (2016). Lactate as a Metabolite and a Regulator in the Central Nervous System. Int. J. Mol. Sci..

[58] Chen S., Minegishi Y., Hasumura T., Shimotoyodome A., Ota N. (2020). Involvement of ammonia metabolism in the improvement of endurance performance by tea catechins in mice. Sci. Rep..

[59] Bush J.A., Wu G., Suryawan A., Nguyen H.V., Davis T.A. (2002). Somatotropin-induced amino acid conservation in pigs involves differential regulation of liver and gut urea cycle enzyme activity. J. Nutr..

[60] Scheiman J., Luber J.M., Chavkin T.A., MacDonald T., Tung A., Pham L.D., Wibowo M.C., Wurth R.C., Punthambaker S., Tierney B.T. (2019). Meta-omics analysis of elite athletes identifies a performance-enhancing microbe that functions via lactate metabolism. Nat. Med..

[61] Liu J., Lkhagva E., Chung H.J., Kim H.J., Hong S.T. (2018). The Pharmabiotic Approach to Treat Hyperammonemia. Nutrients.

[62] Stojanov S., Berlec A., Štrukelj B. (2020). The Influence of Probiotics on the Firmicutes/Bacteroidetes Ratio in the Treatment of Obesity and Inflammatory Bowel disease. Microorganisms.

[63] Evans C.C., LePard K.J., Kwak J.W., Stancukas M.C., Laskowski S., Dougherty J., Moulton L., Glawe A., Wang Y., Leone V. (2014). Exercise prevents weight gain and alters the gut microbiota in a mouse model of high fat diet-induced obesity. PLoS ONE.

